# Developing an identities-based approach to support more robust resilience and recovery in heritage planning and management

**DOI:** 10.1186/s43238-023-00082-4

**Published:** 2023-02-28

**Authors:** Zachary M. Jones, Théodora Pappas

**Affiliations:** DASTU, Politecnico di Milano, Milan, Italy

**Keywords:** identity, heritage, management plans, Matera, resilience, locally based development model

## Abstract

Resilience has become an increasingly important concept in the cultural heritage field, particularly in the aftermath of the unprecedented challenges the COVID-19 global pandemic brought. However, on a conceptual and practical level, resilience remains closely linked to the technical conservation of built heritage, and there remains a need to develop broader approaches inclusive of cultural and socioeconomic components. This article investigates the potential applicability of theoretical concepts linked to identity and identities in heritage planning to help fill these gaps and develop approaches that consider resilience and are better able to address a range of unanticipated disasters. We first review the literature and policy documents to define and identify the potential for identities-based approaches. We then examine the case of Matera, an extremely fragile world heritage site in southern Italy that has been continuously inhabited for more than 8000 years and provides a relevant example of resilience. We explore the trends and effects of globalised tourism development before the pandemic and the post pandemic emergence of more local/regional and slow tourism patterns, largely based on cultural solutions to local development challenges and knowledge exchange. Through this comparison, we analyse the potential and limitations of introducing identities-based concepts into heritage planning as a more robust way to enhance resilience and prepare cities for unexpected future crises.

## Introduction: identity as a link between resilience and cultural heritage

Resilience has become an increasingly important concept in the cultural heritage field. It has been highlighted as a goal to attain or an embedded quality to maintain while recovering from disasters such as the COVID-19 global pandemic, climate change and other crises, such as the conflicts in Syria and Ukraine. The concept of resilience is present throughout the 2021 European Heritage Green Paper, which describes resilience as enabling, enhancing and strengthening cultural heritage in recovery efforts; thus, millions of euros have been directed to the topic, as evidenced by Horizon Europe funding calls. However, the concept of resilience has spread across multiple disciplinary boundaries, and according to some scholars, it has become a mere buzzword and fuzzy concept (Davidson et al. 2016). It is difficult to simply define resilience due to its wide range of usage and intended meanings over time. Walker et al. (2006) proposed to define resilience as “the capacity of a system to experience shocks while retaining essentially the same function, structure, feedbacks, and therefore identity.” Approaches adhering to this interpretation have been largely framed in relation to disaster risk management (DRM) in the preparation of historic sites, landscapes, and practices against future disasters—fires, floods, earthquakes, temperature extremes, etc. Research has thus largely focused on developing various analyses, methodologies, and frameworks to identify vulnerabilities (Ramalhinho and Macedo 2019). However, with the unprecedented shutdown of international tourism and access to cultural heritage sites, the COVID-19 pandemic has revealed a much wider range of threats beyond those posed by natural or man-made disasters (UNESCO 2020a, 2020b). Recent and ongoing military conflicts have also shown that cultural heritage sites remain targets of destruction (Turku 2017), highlighting the urgency of recovery and resilience.

While there is much potential for resilience thinking and approaches to address recovery, there is also a need to further the theorisation of the concept and broaden its implementation. The Shelter Research Project defines resilience as “the ability of an historic urban or territorial system-and all its social, cultural, economic, environmental dimensions across temporal and spatial scales to maintain or rapidly return to desired functions in the face of a disturbance, to adapt to change, and use it for a systemic transformation to still retain essentially the same function, structure and feedbacks, and therefore identity, that is, the capacity to adapt in order to maintain the same identity” (Shelter Project 2019). However, such conceptions of heritage resilience incur the risk of an exaggerated focus on a previous condition that may in fact be less than desirable or on preserving inequalities and injustices in heritage settings (Jones 2022). This risk can be seen unfolding in the post-pandemic situation in which the rush to ‘return to normal’ pre-pandemic levels in the tourism sector comes at the expense of developing new socioeconomic models. This potential risk has highlighted other issues: the complexity of many of the frameworks and methodologies being developed, their ability to effectively capture all relevant aspects of resilience (Stojanovic et al. 2016) and the struggle of practitioners to collect the necessary data to enact these frameworks (Béné et al. 2017). Beyond these issues, the two definitions of resilience cited above make clear that ‘identity’ plays a key role; indeed, identity represents the essence of the item being reinforced and preserved. However, the concept of identity is typically presented as an inherent quality rather than as a possible tool that can be used in making resilience more robust within recovery processes.

This paper offers a new conceptualisation to address the shortcomings that have arisen in the theorisation and implementation of resilience approaches by exploring the potential for the concept of identity and, more specifically, *identities*. Within a systems theory approach, we posit that adopting an identities-based approach in heritage planning and management can serve as a key element in fostering more robust resilience practices over time. As we adopt a widely inclusive and pluralistic approach, we claim that the concept of identity is relevant in addressing the increasing global/local divide, which we see as critical to address the theoretical and implementation issues faced by resilience. Globalisation has greatly impacted heritage in terms of the global recognition and valuing of sites, transfer of expertise and management approaches, growth of mass tourism, effects of climate change and the spread of crises and conflicts (Harrison 2015).

On the one hand, Venice has clearly illustrated several of the effects of the global/local divide and loss of ‘identity’. Mass tourism has made Venice a symbol of many of the negative effects of tourism, as it has been representative of post-industrial development combined with a growing low-cost service sector that has allowed an increasing number of visitors who favour lower quality products and service offers (Grenier 2012). The city has effectively become a “theme-park” (Duquesne 2019), with a product to be consumed, exploiting local resources, identities and territories for touristic aims. The combined impacts of climate change and rising water levels have revealed the extreme fragility of the city to disaster, first as a vulnerable site and second as a place suffering from decades of governmental interference that has been strongly linked to a general vision focused on self-regulated tourism activities (Gravari-Barbas 2018). Despite the extreme efforts made to ensure the protection of the physical heritage of Venice, the disconnect between the understanding of local identities and that of urban and territorial spaces has had profound effects. On the other hand, the ongoing military conflict in Ukraine has revealed not only the vulnerability of tangible and intangible heritage to destruction and attempted erasure but also the role of cultural heritage and identity as a critical resource. Intangible heritage traditions such as the iconic embroidered shirt of the vyshyvanka have already become important symbols of a collective national identity that promotes resilience in the conflict (Lyniv 2022). This shirt and other heritage assets, which support the Ukrainian national identity, will likely play a key role in future recovery and rebuilding processes.

In brief, these examples highlight some of the risks that emerge from a loss of identity, as well as the potential for identity to be activated as a resource. In this paper, we aim to delve into how an identities-based approach could help to better address some aspects of this resource in the management and planning of heritage sites. To do this, we begin by reviewing the theoretical components of an identities-based approach, reviewing the state of the art in the literature regarding the link between heritage and identity and the ways in which this link relates to systems theory and existing conceptions of identity and identities within the concept of heritage. To consider identity in greater detail, we use the case of Matera in Southern Italy to illustrate the plurality of the internal and external identities that have been generated, the ongoing effects of these identities and the potential to be involved in heritage planning and management. While identities-based approaches have not been implemented in Matera, our intention is to reveal the complexity of the identities that are present in place and over time to show how traditional heritage listing, planning and management has failed to fully recognise the breadth and interrelatedness of identities.

Matera is an inherently complex example because it has been continuously inhabited for 8000 years; moreover, Matera’s territory has been developed based on a strong link between nature and culture and closely linked to the concept of identity. Matera has retained a strong sense of local community, largely based on traditions, and has only recently come to interact more directly with globalisation forces of mass tourism, in part due to its selection as a 2019 European Capital of Culture (ECoC). The COVID-19 pandemic halted many of the previously rapidly growing trends in the city, and it is an opportune moment to examine Matera and consider alternative paths forwards away from a simple return to the previous status quo and a turn towards international tourism. Our discussion of Matera derives from two separate investigations that were carried out by the authors and included over 20 interviews with local heritage actors, representatives from the municipality, NGOs, organisers of the 2019 ECoC and local citizens. The interviews primarily provided us with relevant background information to further explore the issue of identity through the subsequent interrogation of secondary sources. We analysed the existing planning documents, heritage reports and evaluations of the ECoC experience. In the final section of the paper, we reflect upon the case of Matera to consider how identities-based approaches could be implemented and used more broadly to work towards a more robust and systems-oriented resilience for cultural heritage. We envision this paper as a first conceptual contribution to lead subsequent research into an exploration of this new approach.

## Grounding the identities-based approach within systems theory

The aim of this section is to broaden the typical understanding of identity within cultural heritage discourses, demonstrating its far greater complexity as well as its ability to complement systems theory approaches and address its relations to established practices. It is beyond the scope of this paper to present a complete theoretical overview of the concept of identity; rather, we refer to discussions coming from other fields to enrich the reflection within cultural heritage. We approach the concept of identity via general system theory (von Bertalanffy 1968), which recognises the continuous interplay of multiple factors and elements rather than isolating them into distinct parts. In his interdisciplinary seminar on identity, Lévi-Strauss argued that the identity question can only be addressed through the lens of complexity by “breaking it down into a multitude of elements,” revealing that no society can take for granted a substantial identity (MacDonald 1978). We start from this definition to question the notion of identity in heritage sites that is often reduced and simplified to fit packaged heritage narratives that are presented for the easy consumption of tourists (Appleyard 1979; Barthel 1996) or that serve as the basis for their heritage listings. Heritage has, of course, long been used to create or buttress national identities—often through a process of dissociation of artefacts, sites or intangible heritage, its original context thus becoming the symbol of a shared imaginary or identity (Elias 1998; Glendinning 2013; Jokilehto 1999; Urry 1995). However, as Ashworth (1994) recognised, local identity can go beyond national or international standardisations to stress truly unique attributes. Identity has clearly long played a pivotal role in the understanding and importance of heritage but has been framed as an inherent, static element rather than a dynamic resource to activate.

### Broadening the concept of identity

The concept of identity has in its own way evolved through contact with globalisation, and the concept has expanded as its links to place have become increasingly complex. Individual conceptualisations of one’s own identity have expanded in reference to diverse forms of belonging, whether these are based on religious, cultural, regional, or associative factors (Kaufmann 2014). Globalisation has contributed to creating an illusion of cultural uniformity through the general perception of the disappearance of borders—real or fictive—and the general reduction in distances in relation to global expanses. However, Foucher (in Foucher and Amilhat-Szary 2020) denounced the danger of this “frontier-free” discourse, largely brought about by global finance and big tech firms. He recalled the importance of borders to separate “the inside and the outside” and create communities as well as a “dialogue” that finds “forms of coexistence” between different groups. At the state and administrative levels, borders then simplify identity according to tangible or measurable elements (e.g., nationality, biological data, etc.) and fail to capture the many elements that make life meaningful to an individual and help to define individuals (Kaufmann 2014). These risks can confound both ideology and identity, the former representing a simplistic promotion of certain social phenomena, while the latter embodies a constantly changing entity in need of reinterpretation (Hinshelwood 2007; Kaufmann 2014).

The term identity expresses a duality, referring both to differentiation and similarity. Given that identity and identification derive from the same logic, identity is always based on a degree of exclusion or individualisation (Ben Jemia 2014), which highlights the importance of including social and territorial components in the definition of identity, as it permits a communal identification process allowing for various groups to be connected. Identity is in that sense found at the centre of an ongoing interaction between subject, society and space (Di Méo 2002). The “subject” refers to three main elements representing “self” expression: an emotional and affective element, a social and cultural aspect and a cognitive dimension. This personal identity relates to a “psychological structure” that evolves through diverse social and individual spatial experiences. This “self” expression is otherwise defined in relation to other people and within a particular environment, that is, in interaction with the society a person relates to, so that personal identity feeds on the ways in which an individual internalises “values”, “ideals” and “standards” emanating from the society to which he or she belongs. In that sense, the territory is a “symbolic field” where identity is expressed, and it is composed of various “signs” that allow this identity to be recognised by the group to which a person belongs. Some of those elements, raised to the rank of “heritage values”, then consolidate the collective identity of the related community (Di Méo 2002). However, the presence of a single community does not necessarily denote the existence of a single identity (Pasqui 2008), and the potential for multiple coexisting or even conflicting identities must be recognised.

### The link between heritage and identity

Focusing on cultural heritage, Fiona McLean (2006) highlighted the lack of discussion about “identity negotiation and construction in heritage”, especially given the “identity-conferring status” of cultural heritage. In fact, she highlighted the case of buildings that have been imbued with undesirable meanings, such as those associated with the Nazi era, and the challenge in presenting them to the public. The question of negative heritage helps us understand the importance of how we decide to consider our past in the present time. According to Macdonald (In McLean 2006, 3), “heritage turns material into ‘identity pasts’ of material that is preserved because of its significance, and that inevitably the heritage effect becomes an identity effect.” The preservation of the past is intrinsically linked to the political, social, and historical values of our present identity construction. Heritage conceptualisations must then include tangible and intangible aspects in relation to local communities and consider how these aspects change over time. Turnpenny (2004, 299), reinforced the link between cultural heritage and identity since “cultural sites, places and artefacts can, therefore, be considered to be physical representations of perceptions of self, community and belonging and their associated cultural values.” According to this view, the management of heritage cities should not be viewed only in terms of maintaining physical protected areas but must also consider the community’s values and identity that are attached to these areas.

The concept of “cultural landscape” perhaps comes closest to expressing the idea of an intrinsic relation between a landscape and the human history that has been recorded in that particular a place. However, this concept has remained largely underrepresented within the listing of the UNESCO’s World Heritage Sites, which could provide an opportunity to pursue a more identities-based approach. In light of the concept of “cultural landscape,” the importance of “culture” as a social construction that is rooted in heritage preservation and has an impact on personal identity can be questioned. In social terms, cultural identity, at the scale of an individual, is acquired by “interaction with [the] group” and by the internalisation of that group’s values into his or her own values (Belgacem 2012). In that sense, cultural identity is not a “stable and definitive state” but a “process” involving “adhesions and identifications” to various ways in which a community acts, is, and thinks; it also involves “oppositions” and “exclusions” relating to “ways of doing”, “being” and “thinking” of neighbouring communities. Culture could then be considered the result of an accumulation of uses in relation to social life and the natural environment; moreover, the natural territory, through its physical existence, expresses the inherited culture of a given place. Hence, heritage is what we select within a culture as having a specific importance; it is key in the representation of what is perceived as important within the collective identity to which an individual relate. Identity is subject to both “internal” and “external” identification processes (Pappas 2021) resulting from social and territorial constructions over time.

### The role of identity in heritage planning and management

The management of historical sites has traditionally been divided between the management of cultural heritage sites and natural heritage sites; moreover, since 2003, intangible heritage sites have been added (UNESCO 1972, 2003). However, this classification system tends to separate cultural, natural and intangible heritage entities rather than recognizing, protecting and promoting their integration. Exploring how identities develop out of the interaction and intersection of these categories rather than in isolation would in fact support UNESCO’s stance that sites cannot be protected “in isolation or as museum pieces”, isolated from social changes, natural or man-made disasters, land-use planning decisions or their communities’ concerns (UNESCO 2013). This realisation supposes the need to seek out the correct balance that avoids preserving heritage based solely on a static understanding and the development of erroneous interpretation of its specific exceptional values (Pappas 2021).

Over the last century, the concept of heritage has expanded from representing a limited number of individual physical elements to including entire urban areas, landscapes and intangible heritage entities (UNESCO 2003). As understandings of heritage have expanded, so have management tools and approaches to preservation and conservation. Historically, the state has traditionally assumed a leading role in terms of sites’ inscriptions, often without consulting local stakeholders (Watremez 2013). As management plans have become required since 2007, we can observe that decentralisation has occurred since local municipalities have taken on an increasingly important role involving greater involvement and participation (Rasoolimanesh et al. 2017). However, globalisation and the growth of the international tourism sector have further complicated or in some cases degraded local people’s sense of identity. In part due to a lack of adequate management and mainly based on “laissez-faire” politics (Gravari-Barbas and Guinand 2017), tourism has had unparalleled impacts on the sociocultural life of people in heritage cities. These effects can be broadly grouped into two trends: the disneyfication and museification of historical centres (Duquesne 2019). The museification of historical centres corresponds to an excessive conservation of the past, which attaches the identity of a place to a single representation of events that occurred at a specific moment in time, as a “showcase” of a bygone past. Instead, the disneyfication process reflects a worldwide global model based on the playful and festive environment originally generated at Disneyland and now duplicated in historic city centres (ibid).

ICOMOS’s *Declaration of San Antonio 1996* pertains to heritage and focuses on the question of identity to the greatest degree, stating that “The authenticity of cultural heritage is directly related to our cultural identity” (ICOMOS 1996). In focusing on the context of the Americas, the document recognises the complex and challenging history of intertwined identities and histories and how these identities contribute to creating cultural heritage, particularly where the population has been displaced, has changed, or has been victim of genocide. The text calls for a move away from selecting or promoting the identity of one group above others and for minority and majority experiences to be equally represented and recognised. Although less recognised than other declarations and recommendations, the *Declaration of San Antonio 1996* provides the clearest basis and support for developing an identities-based approach, which has otherwise not played as significant a role in subsequent documents. The *2011 Historic Urban Landscape* (HUL) recommendation is one such example, where identity is recognised and protected but not necessarily activated. While the importance of local or communal identity is noted in this recommendation, the existence and complexity of multiple identities is not recognised, and there is no exploration into the ways in which identities might play a role as a planning and management tool (Bandarin and Van Oers 2012).

### A new approach to identities in cultural heritage

The literature reveals several issues that emerge as both challenges and opportunities to the development of an identities-based approach. First, the strong tradition of and focus on physical conservatism can itself present a challenge to establishing an equilibrium between complex past identities and present-day individual lived experiences. In heritage cities, an alternative approach could be based on a continuous construction or reflection of identities that integrates both dimensions of an inherited past identity combined with a future vision. Rather than differentiating and prioritizing a single factor, an identities-based approach would need to find this equilibrium between a past heritage and the influence of globalised models while incorporating the interactions between the natural, cultural and intangible elements. Through this process, the identifications of internal and external influences on heritage are recognised; indeed, this process allows us to distinguish between identities that have been generated from within local communities and those that have been projected onto those communities.

Moving from individualisation to systems thinking in heritage management via the concept of identity would also align with new approaches to resilience. Recent research has come to recognise the importance of the social aspect of resilience and of local communities’ involvement within the development of resilience measurement matrices as well as in the decision-making processes themselves (Fabbricatti et al. 2020). This expanded way of thinking about resilience has mirrored wider attempts to use socio-ecological systems (SES) approaches to increase the concept’s overall sustainability (Matin et al. 2018; Partelow 2016) and to position it as a more proactive rather than merely reactive force (Sesana et al. 2019). Hence, the use of systems theory would in fact be a way to address the restrictive aspects of both heritage and resilience that presume and at times assume an improved previous state. Understanding identity as an ever-evolving factor related to many other elements could help to bridge and link the past with the need for future visions. Ultimately, we see an identities-based approach as building upon existing heritage thinking and practice. The *Declaration of San Antonio 1996* provides a strong precedent for this new approach while also complementing the HUL recommendation by providing a new tool to help recognise the inherent complexities of tangible and intangible cultural heritage; both texts work towards a similar end result.

## Exploring identities and identities-based approaches in Matera

In this study, we investigate the case of Matera and its region as a pertinent example to demonstrate the potential of the identities-based approach as a tool to capture a far more complex understanding of the interconnectedness of place and heritage. We use the official UNESCO listing and conduct a broader exploration of the various identities that are present in the city or projected onto it. By no means exhaustive, our intention is to show a more expansive, comprehensive and interconnected understanding of heritage. Until recently, Matera and the wider region of Basilicata lacked strong infrastructural connections with the rest of Italy; indeed, Basilicata has remained one of the least populated and visited regions in Italy. However, due to its perhaps rather specific physical heritage as well as at times troubled history, multiple narratives about the city and its identity have persisted and remained distinctly connected to the local population on a personal level. Traditions have endured in relation to the territory and the rural character of the region; these traditions have revealed people’s attachment to the site both in territorial and social terms, so that members of the local community have been encouraged to participate in the safeguarding of their heritage. However, in recent years, there has been a significant push for the region to open up to global forces, which have tested the boundaries and resilience of local heritage identities. In particular, the 2019 European Capital of Culture programme (ECoC) drew international attention to the city, leading to a dramatic increase in tourism, which doubled in 3 years only (Ponzini et al. 2020). However, the COVID-19 global pandemic caused tourists to vanish overnight while also providing an opportunity to reflect upon emerging narratives and the identities being generated and promoted through these narratives. Following a brief historical overview of the context, we examine more in depth the various internal and external identities that have been created both within Matera and outside of Matera. We aim to demonstrate first the plurality of these identities; second, we aim to show the need for a systems-oriented identities-based approach to ensure the recognition and support of this complex system.

Matera is a city of 60,000 inhabitants located in southern Italy. It is the third oldest continuously inhabited settlement in the entire world following Aleppo and Jericho. It originates in the Palaeolithic era and has been widely recognised for its Sassi, the oldest parts of the city that were excavated out of the rock on which the city is built. These settlements are noteworthy for their sustainable integration within their natural environment and the water collection and distribution system embedded within them (Damiano et al. 1998). In 1948, Matera was named the “shame of Italy” by Palmiro Togliatti, the leader of the Italian Communist Party, for its extreme poverty and poor living conditions, especially within the Sassi area. This new ‘identity’ of the city had dramatic effects, leading to state-led housing plans in the 1950s and 1960s that removed communities from the Sassi and relocated them to modern neighbourhoods to improve their living conditions; these neighbourhoods were spread across the city (Pontrandolfi 2002). While these plans intended to enhance the quality of life for local people, they also left the Sassi abandoned (Mininni and Dicillo 2012); however, many residents have retained the memory of the difficulties of living in the Stassi, and the meaning of the Stassi has endured in spite of the recent transformation of the area.

In the late 1980s and 1990s, the first efforts were made to counter the long-term abandonment of the Sassi through a resettlement programme encouraging people to live in and restore homes there. In 1993, both the Sassi and the Park of Rupestrian Churches were officially listed as World Heritage Sites by UNESCO, creating a significantly new narrative in the city’s development. Subsequently, in 2014, the city was awarded the title of European Capital of Culture for 2019 and began a new tourism-centric phase in the city’s history. One example of this new development trajectory has been the ‘airbnbification’ of the Sassi and city centre, with approximately 25% of available residential spaces being converted to short-term occupation (Picascia et al. 2017). While such developments have greatly helped in reducing abandonment and decay, they have also generated new socioeconomic issues for local residents and have changed the idea that people have of the city through new images and narratives about the Sassi and the city more generally.

### UNESCO listing of universal value

Recognised as World Heritage in 1993, Matera was the first site in southern Italy to be listed, as both the Sassi area and the Park of the Rupestrian Churches were listed (Parco Murgia) for their exceptional value in illustrating diverse stages of human history. The Outstanding Universal Value (OUV) of the sites is based on criteria iii, iv, and v. In the case of Matera, UNESCO recognises the site as follows:

- Criterion (iii): The Sassi and the Park of the Rupestrian Churches of Matera represent an outstanding example of a rock-cut settlement, adapted perfectly to its geomorphological setting and ecosystem and exhibiting continuity over more than two millennia.
- Criterion (iv): The town and park constitute an outstanding example of an architectural ensemble and landscape illustrating a number of significant stages in human history.
- Criterion (v): The town and park represent an outstanding example of a traditional human settlement and land-use showing the evolution of a culture which has maintained a harmonious relationship with its natural environment over time.

The site is currently inscribed in the “cultural site” category within the UNESCO list, despite the predominant presence of natural aspects intrinsically related to the city’s culture and territory. While these criteria describe many of the key features that do indeed make Matera a unique built and natural environment, they do not necessarily capture the more complex and intertwined cultural, social and historical elements that contribute to the city’s more deep-seeded identity. The emphasis on architecture and settlement clearly demonstrates that the conservation practices that may ensure physical resilience have been prioritised, at the expense of the cultivation of an enhanced social and cultural resilience. This standardised values-based approach differs from an identities-based approach, which we address in the following sections.

### The role of literature and film in shaping Matera’s identity from the outside

Even prior to being called a ‘national shame’ following WWII, another external source provided a startling view of the city of Matera and provided the impetus for the drastic changes that would occur in the following decades. In his 1945 book *Christ stopped at Eboli*, Carlo Levi decries the living conditions in the Sassi di Matera, which had been largely ignored by the state and in general cut off from the rest of the country, along with the entire region of Basilicata. The title of the book derives from an expression used by the inhabitants themselves to express their feelings of living in a territory abandoned by God, essentially conveying that not even Christ would enter their territory, instead stopping near the sea at Eboli (near Salerno in the Campania region). This sentiment echoes the reality that Matera and the wider Basilicata region have been in fact completely enclaved, as most of the Mezzogiorno region (the southern part of Italy). However, the book produced a chain reaction mobilising several external entities, with national politicians coming to Matera and discovering for themselves the harsh living conditions of the population; it is on such an occasion that Palmiro Togliatti declared Matera to be a “National shame” in 1948. This declaration led to the identification of Matera as a place of great poverty and a symbol of an Italy divided between a developed north and a poor and mostly rural south. In 1952, the De Gasperi government enacted special law 619 that would eventually cause the Sassi to be emptied of its inhabitants, as they were resettled in new modern collective buildings in the Serra Venerdi, Spine Bianco and San Giacomo neighbourhoods, as well as in the peripheral hamlet called Borgo La Martella. These new areas and communities entailed entirely new ways of living and working that were quite foreign to the former residents of the Sassi, which remained largely abandoned until 1950. Although reflecting extreme circumstances, the ways in which the Sassi were identified by external entities as a degraded and a place unfit for living had significant consequences not only for the people living there but for the entire future direction and development of the city of Matera—in effect ending thousands of years of settlement in the Sassi.

Rubino et al. (2021) noted that cinema had a “saving role” in giving a new image to Matera, largely contributing to the revalorisation and revitalisation of the Sassi, and this well before “politics,” “intellectuals” and the UNESCO nomination did. Cinema’s relationship with Matera began in 1952 with the movie “*Fabbrica dei sogni”*, which actually positively depicted a small enclaved provincial city and its historical richness. Despite this perspective, many other films focused on the misery of the city: *La Lupa* (1953), *Gli Anni Ruggenti* (1962) and *Il Demonio* (1963). These various films expressed the contrasting visions that such external projections can develop or reinforce. In the case of Matera and its Sassi, this confrontation reflected most of the patterns of the period in terms of urban constructions and forms, lifestyles and, more generally, reflected the ways in which people in the north of Italy stereotyped the meridional region. The most famous early film that was set in Matera was Pier Pasolini’s *Il Vangelo Secondo Matteo* (1964), which Mel Gibson would subsequently use again as a model for Jerusalem in *The Passion of the Christ* (2002). Matera also gained international recognition for the opening scenes of James Bond’s *No Time to Die* (2021). However, Foschino (in Rubino et al. 2021) noted that such representations have not necessarily increased the town’s image as a tourism attraction or changed the image of the city. However, Matera’s connection with cinema has constituted a prominent theme, as an international press review that focused on national shame, history, and tourism revealed (Ponzini et al. 2020). These films may have provided some visitors with preconceived conceptualisations of Matera, and visitors may have other locations and histories in mind when visiting the city. Such processes of identification create the risk that the true identify of Matera be inaccurately represented and may have far-reaching consequences, as Carlo Levi’s work had.

### Mixing internal and external identities for the 2019 ECoC programme

The 2019 Matera-Basilicata European Capital of Culture project provided an opportunity for the city to simultaneously invite international talent while also highlighting local cultural excellence. The 2019 Matera-Basilicata Foundation was the autonomous entity responsible for planning and implementing the event in coordination with other local and regional institutions, including the municipality and the sopritendenza for cultural heritage. Early in the process of developing the bid, the Foundation was noticed for involving the local community in generating proposals for events and projects to take place during the year (Jones 2015). However, after being awarded the ECoC title, many residents felt left out of the development process during the preparation years (Ponzini et al. 2020), which resulted in a blended internal/external identity being promoted. This process can perhaps be most clearly seen in one of the two pillars of the project for the ECoC year—the I-DEA exhibition. This exhibition drew on archives and collections spread across the entire Basilicata region, representing varying aspects of the area’s heritage. Five national and international artists were asked to curate a continuously evolving exhibit comprised of these historical objects, images and film footage. This external artistic interpretation of elements representing an internally generated identity received mixed responses and serves as an adequate example of the ways in which international events interact with, promote or even alter the meaning of local issues. The event also led to the restoration and reuse of previously abandoned structures, including Mulino Alvino, a former mill. However, while originally intended to become a museum, the mill was ultimately transformed into a luxury hotel. While the building has been restored and put back into use, it has lost its connection to the territory’s strongly embedded food culture.

Other events focused more on the intangible heritage of Matera, in particular the city’s food tradition. *Mammamiaaa* was a collective project by which a database of thousands of recipes from Materans and visitors alike was created, once again mixing internal and external influences. Other events also highlighted the city’s breadmaking tradition. Meanwhile, the *Atlas of Emotions* presented local traditions and activities as “mapped” by local residents, and this mapping was then assembled into a collective theatrical and interactive experience. This exhibit provided insights into many day-to-day traditions of Materans, covering their local dialect, children’s games and folk tales. Most events were held within the city centre and the Sassi area, with cultural, musical, theatrical and other events taking place in the course of that year in a range of heritage locations. The *Happy Birthday Shame Party* took a festive approach to the city’s former nickname as a way of reclaiming its identity from the external actors who negatively evaluated the city. Despite the focus on the city’s historic core for the hosting of events, event organisers also promoted a new vision for Matera based on the theme of ‘Open Future’, which focused not on the city’s outstanding heritage but rather on Matera as a new, dynamic and creative place (Rotolo 2022). It is too early to know the long-term impact or legacy of the 2019 ECoC programme, particularly in the context of the unanticipated global pandemic that started just a few months after the programme ended in Matera. However, the ECoC title provided an interesting and unique forum in which to observe the intermixing and creation of new identities in Matera, which were generated by both internal and external actors.

### Internal identities in the management of Matera’s heritage

Foschino (2020) evoked the “narrations” associated with Matera and its Sassi, according to him, have made the city into an “exemplary metaphor” of “political and ideological schemes” that do not always represent its factual history. He referred to the former mayor Raffaello De Ruggieri, who portrayed the city as representing “the history of humanity”, on one hand using Matera to address the general history of mankind, while on the other hand erasing the particularity of the city (ibid.). However, residents have taken an active role in “rewriting” the narrative or identity presented by others, particularly those forged by external entities basing themselves on Carlo Levi’s book and that had led to the emptying out and abandonment of the Sassi. Cultural activities have been held in the Sassi neighbourhood since the 1950s when the Circolo Culturale La Scaletta was located there and later strengthened when locals began to return to the Sassi in the 1980s and 1990s, creating new narratives and identities connected to these heritage spaces. The story of the Sassi is primarily a story of people: in Matera, it is these citizens’ initiatives that began to make an initial difference before later ‘official’ efforts such as the 2019 ECoC were introduced. The communitarian character of this heritage and related uses, themselves embedded in the specific territorial components of a local nature, has revealed the plurality of those interdependencies, which can only be represented through a complex systems approach.

As in other places that have chosen tourism for economic development, the risk exists of creating a representation that moves away from the reality of the site, simplifying it through the creation of a “touristic product” to be sold; indeed, the disneyfication process has moved the site away from its complex reality in recent years. One tool working against such forces has been the Matera Site Management Plan (SMP), which has aimed at developing a comprehensive approach to the UNESCO site (Colonna and Fiore 2013). The plan was developed through an understanding of the cultural landscape but did not explicitly follow HUL guidelines. The plan has been noteworthy as its creation relied on the—innovative at the time—implementation of four public workshops in which local residents participated. Three community sessions were held to explore the interactions between heritage and community: ‘Codex/Genetic heritage’; ‘Geoculture and Energies’ and ‘Evolutions and future’. These sessions went beyond the strict definitions of universal value to explore what it means for a community to live in the same place for thousands of years and what the future of the community should be in a world facing unprecedented threats from climate change (Colonna and Fiore 2013). Much of the discussion covered day-to-day activities in Matera, the interactions between people, their territory and intangible heritage elements such as the local food culture. However, the management plan that resulted followed UNESCO’s guidelines to rebuild a working relationship between the state and the managers of the sites and did not include such wider reflections. In addition, due to existing planning regulations, the only planned uses of the Sassi area were limited to residential and commercial activities, reducing potential new uses of these spaces beyond touristic goals. The Matera UNESCO chair at the University of Basilicata was responsible for developing the SMP and called for the historical site of the Sassi and the Park of the Rupestrian Churches to be considered a “continuous cultural landscape”, as together they involved the community and local entities as “knowledge carriers”, along with the various layers of institutional governance (ibid.). Unfortunately, much of the SMP has not been formally implemented following its approval, and most of the proposals deriving from locals have remained mere ideas rather than projects.

However, the rural traditions conserved across the territory have remained an important asset for the city and the region. Local food production and circulation has been important to increase local autonomy and resilience, especially because most peasant communities “often struggle to find the practices of their ancestors again” (Pérez-Vitoria 2020). These practices have been overlooked in favour of modernised agricultural systems, leading to a segmentation between living spaces, production spaces and rural landscapes. This social rural heritage has resurged today in Matera, thanks to local associations such as “*noi ortadini*”—or *Us Gardeners—*whose mission has been to “regenerate abandoned land through natural and sustainable agriculture, to return suburban spaces to the citizens of Matera and make them communal places” (Hubout 2022). This organisation has also aimed at re-educating people about the idea of a circular economy, natural soil regeneration and, more generally, about a conception of agriculture that comes closer to subsistence agriculture, i.e., “diversified and nourishing” (Pérez-Vitoria 2020). This type of agriculture reinvents citizens as “gardeners” who cultivate local products and embody Matera’s ancient agricultural roots. These kinds of bottom-up initiatives have taken a leading role in the face of the local administration’s general rigidity and slowness to adopt new approaches or ideas.

### Linking identities-based approaches and resilience in Matera

In the previous sections, we have examined various approaches to identifying and defining the cultural heritage of Matera through an *identities* lens to highlight the differences between the basis on which the original UNESCO listing was made and broader conceptualisations of heritage. By no means have we intended to present all possible internal and external identities, for it is not possible to do so. Our goal instead has been to demonstrate the wide range of identities-based conceptualisations that have emerged as well as some of the effects—positive and negative—of these understandings. Some of the examples we brought up have already begun to unveil the importance of holistic understandings of identity that link place, territory, nature, society and practices to avoid the exclusive protection of physical heritage artefacts at the expense of wider cultural and social connections. The development of Matera’s SMP has shown that many of the broader social and cultural elements that had been collected through the participation of citizens did not adhere to the standard guidelines set to create the document and were thus left out. While the 2019 ECoC programme led to the restoration of structures such as the Mulino Alvino, the new functions were entirely related to the tourism industry and were no longer meaningfully connected to the area’s intangible food traditions. However, recognising and valuing this intangible heritage as part of Matera’s identity beyond mere physical structures might help to protect connections that are embedded connections in other cultural and social dimensions.

By examining some of Matera’s internal and external identities from Antiquity through the last 80 years, a much richer, complex, interconnected and even subjective view of the city’s heritage begins to emerge in opposition to the identity that is promoted through official listings. This pluralistic approach ensures a much wider range of locally embedded issues that otherwise might be overlooked, including agricultural production, food culture and the intrinsic sustainable integration of built and natural heritage sites. To develop resilience strategies for heritage sites and cities, it is critical to go beyond an exclusive conservation-centric approach and to fully prepare for a range of emerging threats and to help to support recovery; in Matera, it is particularly important to go beyond tourism-centric approaches. For example, an expanded recognition of the kinds of relationships that have existed between spaces, territories and cultural practices in the city’s history would lead to the definition of broader potential reuses of the Sassi spaces rather than being limited by the planning of primarily residential or commercial spaces. Applying concepts derived from public participation would also help to promote the continued use of these practices in the development of future strategies, whereas the failure to put these concepts into practice risks disillusioning locals about the potential relevance of participatory practices. While the participation processes utilised in both the ECoC programme and the development of the SMP have helped demonstrate the range of potential identifications that can be made, they have also highlighted the shortcomings in their activation and implementation. Hence, we propose to use identities-based approaches as tools to complement and achieve many of the goals found in other documents, such as the HUL Recommendation, and to capture the full extent and range of connections between these documents and heritage cities.

## Discussion and conclusions: challenges and opportunities in the development of identities-based approaches

This article has aimed to introduce the concept of an identities-based approach to heritage cities as a tool to develop more complex and robust resilience schemes that can contribute to counter some of the shortcomings of monoculture economies pushed by globalisation. The case of Matera serves to illustrate the potential for adopting systems theory thinking to broaden the concept of a single identity to that of multiple coexisting identities. As noted in the introduction, the COVID-19 pandemic has highlighted the shortcomings of limited resilience approaches that have overlooked the interconnectedness of natural, social, cultural and economic dimensions intrinsically linked to built heritage sites all over the world. The impacts of the pandemic have been strongly felt in Matera; indeed, after several years of consistent growth in the tourism sector and the emergence of a local/global divide, tourism has rapidly declined. The external and internal schemes by which the coexistence of multiple understandings of place and heritage that bring together the built, natural and intangible realms have been highlighted. We posit that this approach can complement existing ways of defining and protecting heritage, such as HUL, by enriching these strategies and helping to maintain the many unique but also changing aspects that define places and the people who live in and interact with these places. In Matera, numerous ‘positive’ and ‘negative’ identity traits have informed and interacted with each other over time. On the one hand, the fact that the city was labelled as the ‘shame of Italy’ drastically affected the city’s development, leading to the abandonment of the historic Sassi area and a long-term negative reputation. On the other hand, this label also gave the impetus for the regeneration of the city and for the creation of new narratives and identities—from the 1993 UNESCO listing to the 2019 ECoC programme and other bottom-up local initiatives seeking to reuse and live in the Sassi. Relying on any one single interpretation or narrative of identity carries great risks and simplifies processes that have taken place to better adapt heritage places to global tourism. A systems-theory approach to identity unveils a more complex set of connections, relationships or even conflicts that exist within places, as well as how these places have changed and continue to change over time.

Increasing consideration for intangible, natural or social aspects in relation to built heritage allows for that heritage to be used as a potential resource for the territory, overcoming the rigidity of models that have framed the historic city only in terms of its past dimension and focused on its physical aspects. However, such approaches can unintentionally lead to heritage becoming a one-dimensional consumer product, failing to capture the complexity of that heritage. Those limitations reduce the capabilities of adaptation and flexibility, leading to greater fragility and reducing the possible resilience levers that might be put into action in the face of shocks. For example, considering tourism as an input into the city’s creativity through knowledge exchanges, rather than as a source of ephemeral and punctual economic value, is more closely aligned with the approach envisioned in SMP participatory processes, in which the “ephemeral citizen” rather than the “tourist” remains in the city.

This article represents only an initial conceptual step, as further work and research are required to develop a clear methodology that can be enacted in other contexts. An identities-based approach focuses on a local context, embracing pluralistic and divergent understandings of identity. There is of course a risk of conflict and disagreement, but this sort of open and transparent discussion is critical to developing more authentic understandings of place and community as well as creating a stronger sense of connection to place. This process requires a regular and ongoing evaluation and interpretation to avoid restrictive understandings attached only to one specific time; instead this process keeps track of changing values and identities. Identifying these key, but often overlooked, aspects of place can in turn lead not only to a better protection of heritage but also to the integration and activation of these potential resources to generate a more diverse set of socioeconomic activities beyond the mere monoculture generated by tourism; this integration would also better respond to risks and generate greater resilience through a more open, adaptable, and inclusive process.

In the future, developing this approach will require us to address several potential challenges, for example concerning the information generated about internal and external identities and the nature and inclusiveness of these processes. Matera embodies the great potential for bottom-up participatory processes in exploring broader connections between heritage and other facets of daily life often overlooked in typical heritage governance and management. However, such processes may not be activated if not supported by local decision makers, heritage experts as well as political and institutional actors. The ways in which these issues are addressed may vary from place to place and should respond to local needs and situations and accompany existing heritage governance and management approaches. There is an ever-increasing need for heritage cities to become more resilient and to be prepared to respond to risks and disasters, which requires ever more complex and intersectional approaches and solutions; therefore, we propose that an identities-based approach can help to meet these goals.

## Data Availability

Not applicable.

## Abbreviations

DRM: Disaster Risk Management
ECoC: European Capital of Culture
HUL: Historic Urban Landscape
ICOMOS: International Council on Monuments and Sites
SMP: Site Management Plan
UNESCO: United Nations Educational, Scientific and Cultural Organization
